# Lycopene Ameliorates Liver Inflammation and Redox Status in Mice Exposed to Long-Term Cigarette Smoke

**DOI:** 10.1155/2021/7101313

**Published:** 2021-11-25

**Authors:** Daniela Fonseca Abdo Rocha, Pedro Alves Machado-Junior, Ana Beatriz Farias Souza, Thalles de Freitas Castro, Guilherme de Paula Costa, André Talvani, Frank Silva Bezerra, Silvia Dantas Cangussú

**Affiliations:** ^1^Laboratory of Experimental Pathophysiology (LAFEx), Department of Biological Sciences (DECBI), Institute of Exact and Biological Sciences (ICEB), Federal University of Ouro Preto (UFOP), 35400-00 Ouro Preto, MG, Brazil; ^2^Laboratory of Immunobiology of Inflammation (LABIIN), Department of Biological Sciences (DECBI), Institute of Exact and Biological Sciences (ICEB), Federal University of Ouro Preto (UFOP), 35400-00 Ouro Preto, MG, Brazil

## Abstract

Cigarette smoke (CS) is the major cause of preventable death worldwide, and it can also cause damage to extrapulmonary organs, such as the liver, mainly due the generation of reactive oxygen species (ROS). The liver is an essential organ for human survival since it is mainly responsible for the body metabolism and among other things and it is the place where many endogenous and exogenous substances undergo biological transformation. Lycopene is a nonprovitamin A carotenoid found in red fruits and vegetables, and its role as a potent antioxidant is well known. In this study, we hypothesized that lycopene could protect mouse liver against long-term CS exposure. Thirty C57BL/6 mice were exposed to twelve cigarette smoke (12 cigarettes per day) for 60 days and pretreated with 25 mg/kg/day or 50 mg/kg/day of lycopene via orogastric gavage. After euthanasia, the hepatic tissue was collected for histopathological, antioxidant defense, oxidative stress, inflammatory, and collagen deposition analysis. Our analysis demonstrated that lycopene results in a suitable outcome to ameliorate the pathological changes, inflammatory and antioxidant profile in a mouse model of long-term CS exposure, and collagen accumulation in the hepatic extracellular matrix. This study demonstrates for the first time that supplementation of lycopene can be a possible pharmacological tool for the treatment of hepatic damage caused by exposure to long-term CS.

## 1. Introduction

The tobacco epidemic represents one of the biggest problems in public health, and it is an important cause of preventable premature death worldwide, killing more than 8 million people per year [1]. Cigarette smoking is the most common form of tobacco used around the world and contains more than 7000 toxic chemicals, including at least 70 carcinogens [2]. These types of substances can cause damage to almost every organ in the human body, including the liver, thus increasing the progression and severity of liver disease [3]. Cigarette smoke (CS) contains many toxic gases, free radicals, and reactive oxygen species (ROS), which in excess can cause oxidative damage to cell constituents and macromolecules, such as membrane lipids, proteins, DNA, and enzymes [2, 4]. Other CS pathogenic mechanisms are related to inflammation and immune changes [5].

The liver is an essential organ with many important functions in the body, including digestion, storage of glucose as glycogen, synthesis of proteins involved in the coagulation cascade, and synthesis and storage of cholesterol, apoproteins, and vitamins. Furthermore, this organ also has a crucial role in the immunity through the action of Kupffer cells (KC), besides being involved in xenobiotic detoxification, including CS, through cytochrome P450 family enzymes [6, 7], since the liver is the central organ of the metabolism. Oxidative stress causes the accumulation of damage cellular macromolecules, which may lead to excessive cell apoptosis, mainly via the mitochondrial pathway, and liver injury [8, 9].

Lycopene is a nonprovitamin A carotenoid and natural potent antioxidant [10] found in red- to pink-colored fruits and vegetables like tomatoes and tomato-based products [11]. Lycopene cannot be synthesized by the human body; it must be consumed through the diet [12, 13]. During the digestion, lycopene particles are released from the food matrix, emulsified, incorporated into chylomicrons, and cleared from the circulation by the hepatocytes via low-density lipoprotein (LDL) receptor-mediated pathway, consequently being stored in the liver, specifically on hepatic stellate cells (HSC) [14, 15].

Lycopene consumption has been associated with minor risk to develop chronic diseases because of its capacity to quench singlet oxygen, inactivate ROS, and extinct free radicals, which is two times more effective than *β*-carotene and ten times more potent than *α*-tocopherol [11, 16, 17]. Furthermore, lycopene can activate the nuclear factor erythroid 2-related factor 2 (Nrf2) signaling pathway [18], a transcription factor that is essential to redox homeostasis [9] and plays an important role against oxidative stress, promoting the expression of antioxidant and detoxifying enzyme genes and repairing organ damage [19, 20].

Our research group has already tested the effects of lycopene administration on lung tissue in the same model of CS exposure [21]. The beneficial effects of lycopene on the lungs were observed previously; we hypothesized that lycopene could also prevent CS damage to extrapulmonary organs, such as the liver. In order to verify the initial hypothesis, we performed histopathological and biochemical analysis on the liver of animals that received lycopene and were exposed to CS. Therefore, this study was aimed at evaluating the possible hepatoprotective role of lycopene in C57BL/6 mice exposed to CS for 60 days.

## 2. Materials and Methods

### 2.1. Animals

Thirty male C57BL/6 mice (8 weeks old) obtained from the Laboratory of Experimental Nutrition, School of Nutrition, Federal University of Ouro Preto (UFOP), were housed under controlled conditions (12 h light/dark, 21°C ± 2°C, 50% ± 10% humidity) with access to food (Nuvilab®, Table 1) and water *ad libitum*. The experimental protocol was approved by the Ethics Committee on Animal Use (CEUA) from the Federal University of Ouro Preto, Brazil (no. 2014/05). The mice were divided into 5 groups (*n* = 6): control group (CG), exposed to ambient air; oil group (OG) received 200 *μ*L of sunflower seed oil (sunflower seed oil from Helianthus annuus, Sigma-Aldrich LTDA) via orogastric gavage; the cigarette smoke group (CSG) was exposed to cigarette smoke; the lycopene plus CS group (L25CSG) and lycopene plus CS group (L50CSG) received 25 and 50 mg/kg of lycopene based on the animal body weight, respectively, and were exposed to cigarette smoke. Lycopene (BASF, LycoVit Dispersion 10%, Germany) was dissolved in sunflower seed oil and was administered via orogastric gavage 12 hours before each cigarette smoke exposure, for 60 consecutive days, according to Campos et al. [22].

### 2.2. Cigarette Smoke Exposure Protocol

The CSG, L25CSG, and L50CSG groups were exposed to 12 commercial full-flavor filtered Virginia cigarettes (10 mg tar, 0.9 mg nicotine, and 10 mg carbon monoxide) per day, 3 times a day (morning, afternoon, and night), in an inhalation chamber (40 cm × 30 cm × 25 cm) with a capacity of 30 L, inside an exhaustion chapel, for 60 consecutive days according to Campos et al. [21]. Each cigarette was coupled to a 60 mL plastic syringe through which the smoke was injected into the inhalation chamber, the burning time of the cigarette was 3 min, and the final smoke generated corresponding to 1 L (3%). The CS and QCSG groups were kept in the smoked chamber for 6 min; then, the lid was removed from the inhalation chamber for 1 min to allow complete exhaustion of the air [23]. The CG and OG were subjected to the same conditions, but without exposure to cigarette smoke. Twenty-four hours after the last cigarette smoke exposure, animals were euthanatized by an overdose of ketamine (130 mg/kg) and xylazine (0.3 mg/kg).

### 2.3. Tissue Processing and Homogenization

Immediately after euthanasia, two liver fragments were collected. The first sample was fixed in 4% neutral-buffered formalin, dehydrated, cleared, embedded in paraffin, cut into 4–5 *μ*m thick sections, and stained with hematoxylin and eosin (HE) and Gomori ammoniacal silver for histopathology. Liver sections stained with Gomori ammoniacal silver were analyzed to characterize the intralobular collagen deposition. Then, 100 mg of the other part of the hepatic tissue was placed in polypropylene tubes with 1 mL of phosphate buffer (pH 7.8) and it was homogenized in a tissue homogenizer. Subsequently, the samples were centrifuged at 4°C for 10 minutes at 13.000 rpm (MIKRO 200R; laboratory technology Hettich, Tuttlingen, Germany); then, the supernatant was collected and stored at -80°C and later used for biochemical analyses [24].

### 2.4. Morphometric and Stereological Analysis

Twenty random pictures were taken both in HE and Gomori ammoniacal silver stains using a Leica DM500b optical microscope coupled to Leica DFC 300FX digital camera and digitized by Leica Application Suite V 3.6 Software in a 40x objective. In HE-stained sections, degenerative processes were measured, detected, and graded by semiquantitative assays, such as hydropic degeneration, sinusoidal congestion/hyperemia, inflammatory reaction, intralobular granuloma, hemorrhage, and necrosis.

The changes in the experimental histopathological parameters present were semiquantified according to a scoring method as follows: (0) absent; (1) mild, when present in approximately less than 33% of the total area of the tissue; (2) moderate, when present between approximately 33% and 66% of the total area of the tissue; and (3) intense, when present in approximately more than 66% of the total area of the tissue [24, 25].

In order to evaluate the number of total inflammatory cells in the hepatic lobes, the resident Kupffer cells were included and the ImageJ/Fiji 1.46r program was used (Wayne Rasband, National Institute of Mental Health, Maryland) in a total area of 370895 *μ*m^2^. After, twenty-five random pictures were taken using the light microscope from the Laboratory of Experimental Pathophysiology (LAFEx, UFOP) equipped with a digital camera Axiocam 105 (Carl Zeiss AG, Oberkochen, Germany) attached to the ZEN lite image capture software, utilizing a 100x magnification with immersion oil for differential mononuclear and polymorphonuclear cell counts, based in the counting of 100 cells. There were two different domain researchers who counted the slides at different times by double-bind counting [21].

The collagen fiber deposition in the livers was evaluated in a cycloid test system attached to a monitor screen, where each collagen fiber was assessed by a test point system, which consists in 16 points and a known area in which the boundary line was considered forbidden to avoid an overestimation in the number of structures. This system objective is to evaluate the volume density of collagen fibers (Vv[col]) in the hepatic tissue, as described for the same group previously [26]. The number of the points (Pp) that touched Vv[col] was assessed according to the total number of test points (Pt) in the system by using the equation Vv = Pp/Pt [27].

### 2.5. Antioxidant Defense and Oxidative Stress Biomarkers in Hepatic Tissue Homogenates

The hepatic homogenates were used to assay catalase (CAT) and superoxide dismutase (SOD) activities (U/mg protein). CAT activity was measured according to the Aebi [28] method. Hydrogen peroxide (H_2_O_2_) decomposition was calculated using a molar absorption coefficient of 39.4 M^−1^ cm^−1^. Superoxide dismutase (SOD) activity was measured as described by Marklund S. and Marklund G. [29], according to the capacity of SOD to inhibit pyrogallol autoxidation. In order to evaluate total glutathione content in the hepatic tissue homogenates, an assay adapted from a commercial kit (CS0260, Sigma, St. Louis, MO) was used. It is based on a kinetic method that measures the total levels of glutathione (GSH + GSSH). In this assay, the reduction of DTNB (5,5′-dithiobis-(2-nitrobenzoic acid)) to TNB (5-thio-2-nitrobenzoic acid) was read at an absorbance of 570 nm [30]. For glutathione oxidized (GSSH) assay, the same assay kit was used and 4-vinylpyridine (Sigma-Aldrich Co) was added to the hepatic tissue homogenates for derivatization. If the concentration of GSSH is subtracted from the total glutathione concentration, the concentration value of reduced glutathione (GSH) is provided and the GSH/GSSH ratio is obtained by dividing both concentration values.

Lipid peroxidation was determined by thiobarbituric acid reactive substance (TBARS) (nmol/mL protein) assay [31]. 500 *μ*L of the supernatant from hepatic tissue homogenization was mixed in trichloroacetic acid (TCA) and TBA, and the precipitates were removed by centrifugation at 10000 rpm for 10 min at 4°C and were read at an absorbance of 532 nm [32]. Protein carbonylation (nmol/mg protein) content was assessed according to the Reznick and Packer [33] method, and the supernatant absorbance was read at 370 nm.

### 2.6. Enzyme-Linked Immunosorbent Assay (ELISA) for Inflammatory Mediators

Hepatic tissue homogenates were used to determine the concentrations of the IL-6, TNF, and IL-10 inflammatory mediators. The samples from each group were thawed, and excess proteins were removed by acid/salt precipitation, as previously described [34]. Briefly, equal volumes of the homogenate and 1.2% trifluoroacetic acid/1.35 M NaCl were mixed and incubated at room temperature for 10 minutes followed by centrifugation for 5 minutes at 10000 rpm. The supernatant salt content was adjusted to 0.14 M sodium chloride and 0.01 M sodium phosphate at pH 7.4 prior to determination of the concentrations of IL-6, TNF, and IL-10 using commercially available ELISA kits (BioSource International, Inc., CA, USA) according to the manufacturer's guidelines.

### 2.7. Statistical Analyses

The sample size was calculated using a statistical power test of 95% and a significance level of 5% (BioEstat 5.3) in the pilot study. The variable used to calculate power was superoxide dismutase activity. The normal distribution of each variable was assessed using the Kolmogorov-Smirnov test. The homogeneity of the variances was evaluated by Bartlett's test. For comparison between groups, parametric data were analyzed using the one-way ANOVA test followed by the Bonferroni posttest. Data were expressed as mean ± standard deviation. For discrete data, the Kruskal-Wallis test was used followed by Dunn's posttest and expressed as median, minimum, and maximum values. The difference was significant when *p* < 0.05. All tests were performed with GraphPad Prism version 8.4 (GraphPad Software; San Diego, CA, USA) for Windows 10.

## 3. Results

### 3.1. Histopathological Analysis of the Liver Parenchyma

**Figure 1:**
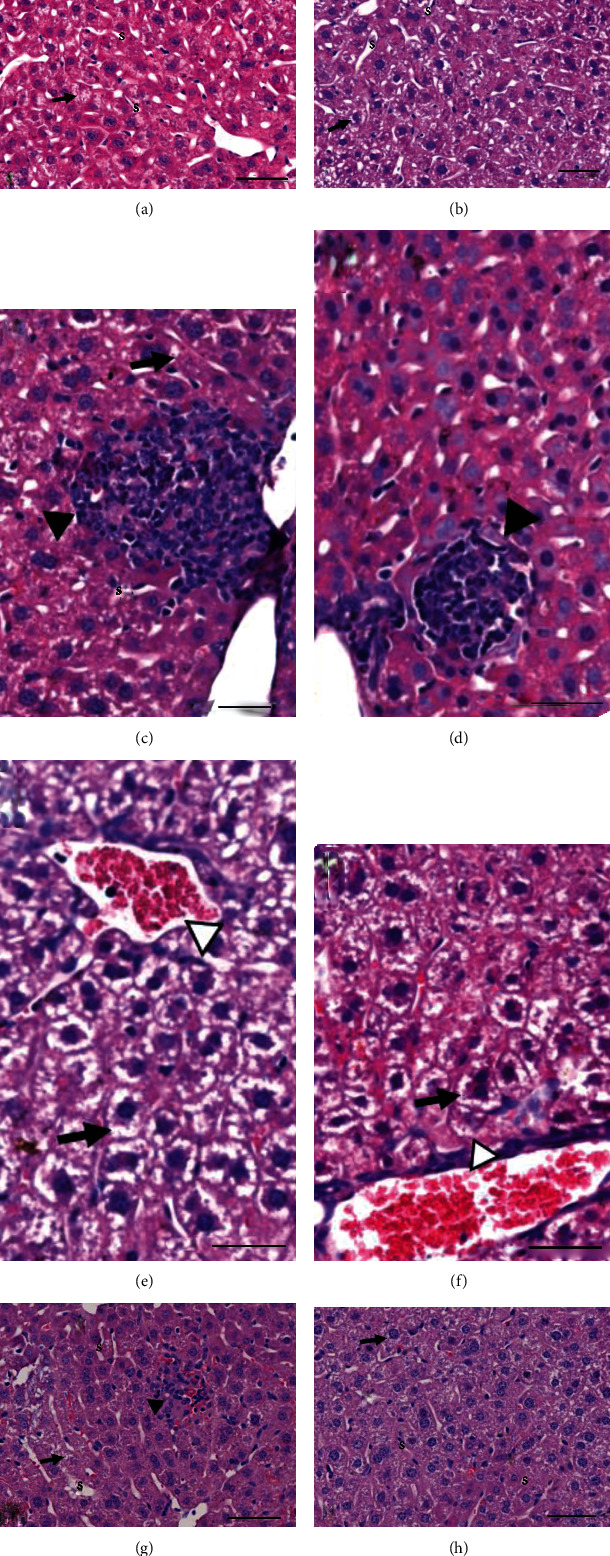
Histopathological aspects of the liver. Stain: HE. Bar = 50 *μ*m. 400x magnification. (a) Control group (CG) and (b) oil group (OG), preserved liver parenchyma, presence of hydropic degeneration (black arrow), and preserved sinusoid capillaries (S). (c–f) Lesions found in the group exposed to CS (CSG). (c) Big area occupied by inflammatory reaction (black arrowhead), hydropic degeneration (black arrow), and preserved sinusoid capillaries (S). (d) Granulomatous reaction detail (black arrowhead). (e) Sinusoidal congestion/hyperemia (white arrowhead and hydropic degeneration (black arrow). (g) Group pretreated with lycopene 25 mg/kg/day (L25CSG), hydropic degeneration (black arrow), inflammatory reaction (black arrowhead), and well-preserved sinusoid capillaries (S). (h) Group pretreated with lycopene 50 mg/kg/day (L50CSG), hydropic degeneration (black arrow), and well-preserved sinusoid capillaries (S).

The histological assays revealed that CG had almost completely preserved hepatic histology (Figure 1(a), Table 2), such as OG (Figure 1(b), Table 2). Tissue morphological analysis in the CSG had evidenced injuries, such as lobular and portal inflammatory reaction, granulomatous reaction, and sinusoidal congestion/hyperemia (Figures 1(c)–1(f), Table 2). Lycopene administration (25 mg) resulted in lower, but not total, CS injuries disappearing (Figure 1(g), Table 2). Lycopene administration (50 mg) showed better results relative to CS hepatic injuries (Figure 1(h), Table 2).

Hydropic degeneration was observed similarly in all groups (Figures 1(a)–1(h), Table 2). Sinusoidal congestion/hyperemia was observed in the livers of all groups; however, CSG had more advanced stages and large distribution within the liver parenchyma of these damages (shown in Figure 1(d), Table 2). Furthermore, lycopene administration in both concentrations reduced the intensity of these lesions (Table 2).

Semiquantitative analysis showed inflammatory reaction in the liver of CSG (Figures 1(e) and 1(f)). There was not any inflammatory reaction in the CG; only resident inflammatory cells were observed (Figure 1(a)). Lycopene administration (25 mg) has not been able to significantly reduce the inflammatory reaction (Figure 1(g)). However, L50CSG showed similar statistics to CG and OG (Figures 1(a), 1(b), and 1(h)).

Granulomatous reaction was observed only in the CSG (Figure 1(d), Table 2). Consequently, there was significant difference when the CSG was compared with all other groups (Table 2). Necrosis and hemorrhage focus were not observed in the experimental groups (Table 2).

The number of inflammatory cells to the liver was greater in the CSG compared to CG (Table 3). The pretreatment with lycopene (25 mg) did not show a beneficial effect about this parameter. On the other hand, lycopene pretreatment at 50 mg was effective to significantly reduce the number of inflammatory cells (Table 3). The differential inflammatory cell count showed predominant mononuclear inflammatory in the hepatic tissue. CSG had a greater number of mononuclear cells compared to CG, and only the pretreatment at 50 mg was effective. Polymorphonuclear cell count did not show any difference between the groups (Table 3).

**Figure 2:**
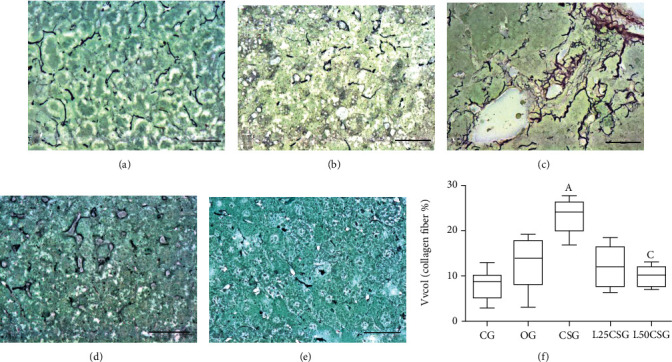
Hepatic collagen deposition. Stain: Gomori ammoniacal silver. Bar = 50 *μ*m. (a) Control group (CG). (b) Oil group (OG). (c) Group exposed to CS (CSG). (d) Group pretreated with lycopene 25 mg/kg/day (L25CSG). (e) Group pretreated with lycopene 50 mg/kg/day (L50CSG). (f) Volume density (Vvcol) of collagen fibers. Data were expressed as mean ± SD. Statistical analysis: one-way ANOVA test followed by Tukey's posttest. A represents a significant difference between CSG and CG; C represents a significant difference between CSG and L50CSG, *p* = 0.003.

Collagen fiber deposition was morphologically evident in the CSG, but not in the other groups (Figures 2(a)–2(f)), and only the pretreatment with 50 mg/kg/day of lycopene (L50CSG) showed a decrease in the volume density (Vv[col]) of collagen fibers.

### 3.2. Antioxidant Defense and Oxidative Stress Biomarkers

**Figure 3:**
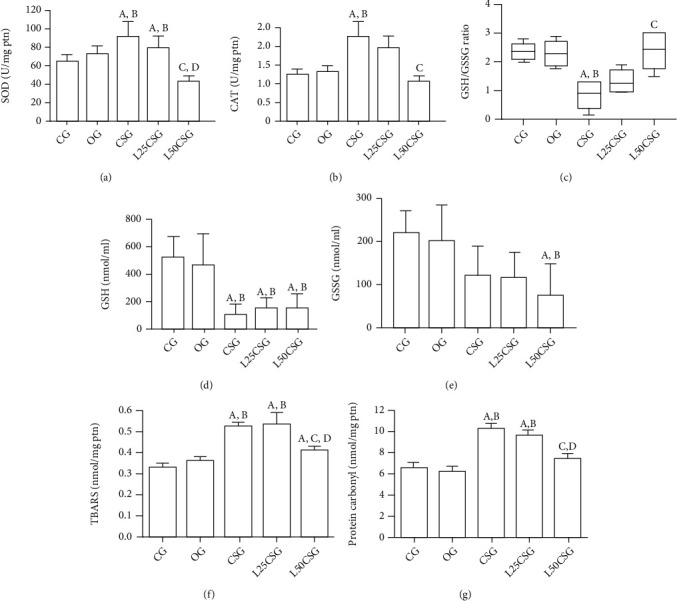
Antioxidant defense and oxidative stress biomarkers in the liver. (a) Superoxide dismutase (SOD) activity in the liver; (b) catalase activity in the liver; (c) GSH/GSSG ratio; (d) reduced glutathione (GSH); (e) oxidized glutathione (GSSG); (f**)** levels of thiobarbituric acid reactive substances (TBARS) in the liver; **(**G**)** carbonylated protein levels in the liver. CG: control group; OG: oil group; CSG: cigarette smoke group; L25CSG: lycopene 25 mg/kg/day and cigarette smoke group; L50CSG: lycopene 50 mg/kg/day and cigarette smoke group; GSH: glutathione sulfide; GSSG: oxidized glutathione. A represents a significant difference when compared to CG; B represents a significant difference when compared to OG; C represents a significant difference when compared to CSG; D represents a significant difference when compared to L25CSG. SOD, CAT, TBARS, and protein carbonyl data were expressed as mean ± standard error of the mean and were analyzed by one-way ANOVA followed by Tukey's posttest (*p* < 0.05). The GSH/GSSG ratio data were expressed as median, minimum, and maximum value and were analyzed by the Kruskal-Wallis test followed by Dunn's posttest, *n* = 6 animals per group (*p* < 0.05).

CSG has higher SOD activity compared to CG and OG, which was attenuated only by pretreatment with lycopene at 50 mg. Likewise, increased catalase activity was observed in the CSG compared to the CG and OG. However, there was a reduction of the enzyme activity in the L50CSG compared to CSG. The GSH/GSSG ratio was lower in the CSG than CG and OG. In the administration of lycopene, only the concentration of 50 mg increased the GSH/GSSG ratio compared to CSG. TBARS level was higher in the CSG compared to CG and OG, and the pretreatment with lycopene 50 mg was able to reduce this parameter. Regarding protein carbonyl, its levels in the CSG were higher when compared to those in CG and OG, and only lycopene 50 mg reduced it (Figure 3).

### 3.3. Levels of Inflammatory Mediators in the Liver

**Figure 4:**
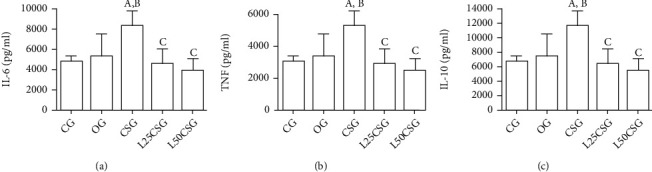
Inflammatory markers in the liver. (a) Interleukin 6 (IL-6) levels in liver homogenate. (b) Tumor necrosis factor alpha (TNF-*α*) levels in liver homogenate. (c) Interleukin 10 (IL-10) levels in liver homogenate. CG: control group; OG: oil group; CSG: cigarette smoke group; L25CSG: lycopene 25 mg/kg/day and cigarette smoke group; L50CSG: lycopene 50 mg/kg/day and cigarette smoke group. A represents a significant difference when compared to CG; B represents a significant difference when compared to OG; C represents a significant difference when compared to CSG. Data were expressed as mean ± standard error of the mean and analyzed by one-way ANOVA followed by Tukey's posttest, *n* = 5 animals per group (*p* < 0.05).

CSG showed higher levels of TNF-*α*, IL-6, and IL-10 in liver homogenate when compared with CG and OG. For all markers, pretreatment with lycopene in both concentrations, 25 mg/kg/day and 50 mg/kg/day, led to a decrease in cytokine levels compared to CSG (Figure 4).

## 4. Discussion

In this study, we observed that both doses of lycopene were able to improve the effects of CS exposure in hepatic injuries, confirming our initial hypothesis. We observed injuries in the hepatic parenchyma and redox imbalance provoked by CS exposure, confirming the results of a recent study performed by our research group with the same CS mouse-exposure model [24]. Despite the scientific evidence that supports the beneficial effects of lycopene on liver parenchyma [17, 35], this is the first study that assesses the hepatoprotective role of lycopene in an experimental model of exposure to long-term CS.

Previously, our research group demonstrated using the same CS exposure model that lycopene is an antioxidant and an anti-inflammatory agent in the lung by reducing oxidative stress and inflammatory response in a short- [16] and long-term exposure to CS [21]. In the mice exposed to long-term cigarette smoke, there was a decrease observed in the body weight of CSG, L25CSG, and L50CSG groups at the end of the experiments, when compared to the beginning [21]. This result shows that lycopene did not alter the body weight of the animals, even though the animals were administered orogastric sunflower seed oil. On the contrary, their weight decreased and it is probably an effect of the CS, which may increase animals' metabolic rate and decrease their appetite [36].

The liver plays a fundamental role in the mammalian energetic balance. Lycopene is a natural carotenoid found in tomatoes and their products, and it exhibits potent antioxidant capacity, which may protect the liver against damage [11, 37]. Recently, our research group demonstrated that lycopene exerts an anti-inflammatory role against hepatic lesions induced by acetaminophen (APAP) in C57BL/6 mice, improving the redox state [17]. Similarly, another study *in vitro* and *in vivo* performed by our group showed that lycopene reduced the production of ROS in SK-Hep-1 cells [38].

We observed that both doses of lycopene were able to improve the effects of CS exposure in injuries, such as sinusoidal congestion, hyperemia, and granulomatous reaction, which decreased or disappeared. Only the dosage of 50 mg/kg was able to reduce the inflammatory reaction. One study performed with methotrexate-induced liver injury in rats also described that lycopene at 10 mg/kg/day was able to prevent congestion and sinusoidal dilatation [39].

The great influx of inflammatory cells in CS groups is probably due to the release of ROS, which led to the activation of resident macrophages, the release of proinflammatory cytokines, and the recruitment of inflammatory cells [40]. The predominance of mononuclear cells in animals exposed to CS suggests an increase in the number of activated KC and the recruitment of macrophages, lymphocytes, and Natural Killer (NK) cells [40, 41].

Previous studies have demonstrated an increase in the deposition of collagen fibers and/or an induction of hepatic fibrosis associated with CS in animal models [42]. In our study, the long-term CS exposure significantly increased the deposition of collagen fibers and normal deposition was restored by the administration at 50 mg of lycopene. Probably, this carotenoid reduced oxidative stress caused by cigarette smoke and the activation of hepatic stellate cells (HSC) to myofibroblast, most likely via activation of TGF-*β*1, a profibrotic cytokine [15, 43].

Previous data showed higher levels in the antioxidant enzyme activities and promotion of oxidative damage in CS exposure models [44]. Our results showed that the cigarette smoke significantly increased the activities of CAT and SOD, and the lycopene pretreatment at both doses was effective in preventing the reduction of SOD activity. However, only the lycopene pretreatment at 50 mg was able to reduce CAT activity. This modulation of antioxidant enzyme activities is probably due to the lycopene ability to suppress the singlet oxygen [11].

GSH is a potent intracellular antioxidant that plays a role in the detoxification of electrophilic compounds and protects the body against oxidative damage [45]. During the detoxification of peroxides, GSH is oxidized in GSSG; this reaction is catalyzed by Glutathione Peroxidase (GPx), and a decrease in GSH/GSSG ratio is an evidence of cellular redox state [46]. Our results indicated that CS decreased the GSH/GSSG ratio, which suggests an increase in the oxidation of GSH and high GPx activity. We also found that the GSH/GSSG ratio was reduced in a recent study by our research group with the same experimental design [47]. On the other hand, the enzyme glutathione reductase (GR) catalyzes the reduction of GSSG to GSH, reconstituting GSH homeostasis [48]. Under stress conditions such as CS exposure, the nuclear factor erythroid 2-related factor 2 (Nrf2) is translocated to the nucleus and induces the transcription of some cellular defense genes, such as GSR [49]. Despite that, we did not evaluate nuclear translocation of Nfr2; lycopene is well known to activate the Nrf2 pathway [18], which may contribute to the increase of GSR levels and GSSG reduction into GSH. This fact justified the increase of GSH/GSSG ratio in L50CSG when compared to CSG, reinforcing the lycopene hepatoprotective role.

The CS is known to cause oxidative damage to macromolecules [50]. The CS exposure led to an increase of lipid peroxidation, and only the pretreatment with lycopene 50 mg/kg/day was capable of reducing it. One previous study, also from our research group, showed that lycopene 10 mg/kg/day modulates lipid and lipoprotein metabolism damage caused by oxidative stress in liver tissue induced by APAP overdose [17]. Our results demonstrate that lycopene 50 mg/kg/day was effective in decreasing lipid peroxidation and in preventing oxidative damage in the liver parenchyma of CS-exposed mice.

The group submitted to CS had higher levels of oxidized proteins when compared to CS and OG. Our results corroborate with those found by Sato et al. who also observed protein oxidation in the livers of mice exposed to cigarette smoke [51]. Only the supplementation with 50 mg/kg/day of lycopene was able to prevent the oxidation of protein and lipids in the livers of mice. In a study by our research group in the model of intoxication with APAP, both concentrations of lycopene (10 mg/kg and 100 mg/kg) were effective in reducing protein carbonyls [38].

We observed an increase of IL-6, IL-10, and TNF-*α* in the C57BL/6 mice exposed to CS. Lycopene pretreatment at both doses was able to reverse the increase of the cytokines. Mechanisms behind how CS affects the immune system are probably related to the ROS production, which upregulates the expression of some cytokines, such as IL-6 and TNF-*α* [41], both produced by macrophages, which matches with the increasing of mononuclear cells as described above. Furthermore, ROS can also enhance the nuclear factor kappa B (NF-*κ*B), a transcription factor involved in the inflammatory process to induce proinflammatory cytokine production, and lycopene is well known to suppress the NF-*κ*B signaling pathway [15, 52]. Our results are supported by other studies that did observe an increase of the pro- and anti-inflammatory cytokines in the liver parenchyma of different CS exposure models [24, 53]. We believe that the increase in the IL-10 levels is probably a balance response to protect the host against the damage caused by the release of proinflammatory cytokines.

Thus, we believe that lycopene worked to improve the hepatic injury induced by cigarette smoke in different signaling pathways and that lycopene has a promising role as an auxiliary pharmacological tool in the treatment of damage to the liver due to the exposure to CS. However, the findings of this study must be seen in light of some limitations. In our experimental model, we performed the pretreatment with lycopene, which is different from what happens in reality. In addition, It was not possible to measure the activity of the liver enzymes alanine aminotransferase (ALT) and aspartate aminotransferase (AST), both important parameters of liver injury [54]+, despite the lack of association of smoking with ALT and AST reported in most studies [54, 55]. The third limitation was concerned with the measure of the inflammatory mediator TGF-*β*1, an important fibrogenic mediator [56]. Finally, in our experimental model, we performed the pretreatment with lycopene, which is different from what happens with smokers. Further studies evaluating the mechanisms of the pharmacological action of lycopene, as well as the dose required in humans to observe hepatoprotective effects and thus determine its use as a therapeutic agent in the pretreatment of liver injuries, are needed.

In conclusion, this study showed that lycopene was effective in preventing liver damage caused by long-term exposure to CS; consequently, it has limited histopathological injuries, as well as the recruitment of inflammatory cells and collagen deposition.

## Figures and Tables

**Table 1:** Composition of the experimental diet (g/1000 g of diet).

Nutrients (g)	Standard diet
Carbohydrates	657
Protein	193
Fat	80
Fiber	10
Mineral mix^a^	50
Vitamin mix^a^	10
Energy density (kcal)	4220

^a^Vitamin and mineral mix following the Ain-93M recommendation for rodents.

**Table 2:** The semiquantitative score values (minimum-maximum) of the histological lesions found in the liver.

Lesions	CG	OG	CSG	L25CSG	L50CSG
Hydropic degeneration	2 (1-3)	2 (1-3)	2 (1-3)	1 (1-2)	1 (1-3)
Sinusoidal congestion/hyperemia	0 (0-1)	0 (0-1)	3 (2-3)^a,b^	1 (0-1)	1 (0-1)^c^
Inflammatory reaction	0 (0)	0 (0-1)	2 (1-3)^a,b^	1 (0-1)	0 (0-1)^c^
Granulomas	0 (0)	0 (0)	1 (0-1)^a,b^	0 (0)^c^	0 (0)^c^
Bleeding focus	0 (0)	0 (0)	0 (0)	0 (0)	0 (0)
Necrosis	0 (0)	0 (0)	0 (0)	0 (0)	0 (0)

CG: control group; OG: oil group; CSG: cigarette smoke group; L25CSG: lycopene 25 mg/kg/day and cigarette smoke group; L50CSG: lycopene 50 mg/kg/day and cigarette smoke group. a represents a significant difference between CSG when compared to CG; b represents a significant difference between CSG when compared to OG; c represents a significant difference when compared to CSG. The data were analyzed by Kruskal-Wallis followed by Dunn's posttest, *n* = 6 animals per group (*p* < 0.05).

**Table 3:** Number and types of inflammatory cells found in the liver.

	CG	OG	CSG	L25CSG	L50CSG
Total (cells/total area)	62.36 ± 5.36	56.26 ± 6.34	92.77 ± 14.80^a,b^	77.61 ± 10.17^b^	62.61 ± 12.07^c^
Mononuclear (cells/total area)	57.02 ± 6.11	52.00 ± 4.59	84.02 ± 14.53^a,b^	71.79 ± 8.42^b^	57.98 ± 11.83^c^
Polymorphonuclear (cells/total area)	5.34 ± 1.82	4.26 ± 2.56	8.75 ± 5.32	5.82 ± 1.91	4.63 ± 1.62

CG: control group; OG: oil group; CSG: cigarette smoke group; L25CSG: lycopene 25 mg/kg/day and cigarette smoke group; L50CSG: lycopene 50 mg/kg/day and cigarette smoke group. a represents a significant difference between CSG when compared to CG; b represents a significant difference between CSG when compared to OG; c represents a significant difference when compared to CSG. The data were expressed as mean ± standard deviation and were analyzed by one-way ANOVA followed by the Tukey posttest, *n* = 6 animals per group (*p* < 0.05).

## Data Availability

The data obtained in this study are available from the corresponding author upon request.
